# Integration of the Verbatim Exercise into a Hospice and Palliative Medicine Fellowship

**DOI:** 10.1089/pmr.2022.0025

**Published:** 2023-05-02

**Authors:** Christopher R. Powers, Garrett E. Snipes, Katie Boykin Harbin, Andrew Fischer, Nancy Anderson, Kevin Cheng, Kristi Ford-Scales, Bryan C. Siefert

**Affiliations:** ^1^ Spartanburg Regional Medical Center, Spartanburg, South Carolina, USA.; ^2^ Novant Health Presbyterian Medical Center, Charlotte, North Carolina, USA.; ^3^ AuthoraCare Collective, Greensboro, North Carolina, USA.

**Keywords:** fellowship education, mindfulness, resiliency, self-care, verbatim

## Abstract

One of the more challenging aspects of specialty level training in any medical fellowship is learning to communicate mindfully and effectively with patients and families in the face of serious illness. For the past five years, our accredited Hospice and Palliative Medicine (HPM) fellowship program has been integrating the “verbatim”—an exercise with a long and integral history in the training of health care chaplains. Verbatims are word-for-word accounts of a clinician's encounter with a patient and/or the patient's family. The verbatim serves as a formative educational exercise offering a method to hone one's clinical skills and competencies, while providing a unique space to practice self-awareness and self-reflection. Although sometimes difficult and intense for the fellow, we have found this to be a helpful exercise in improving the fellow's ability to make meaningful connections with patients and leading to improved outcomes of communication episodes. This potential growth in self-awareness supports both resiliency and mindfulness, which are essential skills for longevity and reduction of burnout risk in the field of HPM. The verbatim invites all participants to reflect on their own participation in facilitating whole person care to patients and families. Out of the six HPM milestone metrics for fellowship training, the verbatim exercise supports achievement in at least three of the milestones. We present survey data over the past five years of our fellowship in support of the utility of this exercise and for consideration to include this exercise in a palliative medicine fellowship. We offer additional suggestions for further study of this formative tool. This article delineates the verbatim technique and its specific integration into our accredited ACGME Hospice and Palliative Medicine fellowship training program.

## Introduction

The verbatim is a commonly used exercise in clinical pastoral education.^1^ The verbatim exercise dates to the 1920s^2^ when physicians and chaplains working together adopted and implemented this study.^3^ Although it has remained an integral part of health care chaplaincy training for many decades,^3^ it is surprisingly not widely found in formal use in medical education. A current review of the medical literature finds no articles addressing the integration of the verbatim exercise into any form of medical education with the exception of health care chaplaincy.

Verbatims are recollected and written word-for-word accounts of a clinician's encounter with a patient and/or family members. The writer of the verbatim is directed to include a reflective response on the presented encounter that identifies both their felt emotions and any memorable challenges experienced. The verbatim is then discussed with both the trainee and trainers experienced in working with verbatims. The verbatim trainers (facilitators) also participate in the verbatim review with a trainee while providing constructive and supportive critique. The verbatim exercise offers an opportunity for a trainee to more deeply reflect upon clinical conversation skills and feelings and emotions experienced within.

The verbatim in a palliative care context invites a physician trainee to be more than just prescriptive, to do more than just give directions or recommendations. Communication in palliative medicine conversations must have dialogue and an exchange of ideas or opinions on a particular issue. Participation in the verbatim exercise may potentially help promote progress in achievement of some of the six recommended ACGME Hospice and Palliative Medicine (HPM) fellowship milestone competencies, including interpersonal communication, practice-based learning, and professionalism^4^ while providing the opportunity to support the practice of self-awareness for the fellow, especially during challenging palliative care conversations.

Since 2017, our accredited HPM fellowship teaching faculty took the unique opportunity of implementing the verbatim as a formative educational resource to enhance the educational needs of our HPM fellows.

To more fully demonstrate its efficacy, we present an example verbatim and a reflection and analysis from a former HPM fellow. We also present survey data from five former fellows. We suggest further research and the invitation to integrate this educational tool to enhance learning and promote improved competency, wellness, and resiliency in difficult palliative care conversations.

## Methods

Verbatim account excerpt. See Table 1.

**Table 1.** Verbatim Account Excerpt

**Disclaimer: All identifiable information about the patient and family has been removed and/or further de-identified to protect both the patient and the family.**
*The family meeting began that day in a family consultation room with the patient's spouse (S), 3 adult children (C1,C2, and C3) and 1 grandchild on a smart device. p-* = *the palliative care fellow physician. P1–P8, the sequential statements of the fellow.*
***There was tension in the room from the beginning…***
*P1: Ms. Z is continuing to decline despite the critical care team's best efforts. Her vital organs are now failing her, and I worry that she will not survive this hospitalization.*
*S: I don't believe that. I know friends who have survived death sentences given by doctors. I knew a person in the military who is now a paraplegic after a major accident. The doctors told him that he would die because he had blood on his brain. He spent many months, many months (!), on a ventilator before getting out of the hospital and guess what? He's still alive today using a wheelchair.*
*C1: You know she's previously survived a life-threatening illness, so she can survive anything. I think you and that other ICU doctor are wrong. I am praying for everybody in that hospital every day. You will find something to cure her. With all the other doctors taking care of her, I know you all will find something.*
*P2: Given the severity of her illness, I worry that she will not be able to recover to…*
*C1: Negative negative negative. That's all we hear from the doctors. The nurse yesterday said that she is doing good.*
*S: We know miracles are possible. I've seen them. And we know a miracle is going to happen here.*
*P3: In this situation, what miracle would you hope for?*
*C2: She's going to wake up and walk out of that hospital. You watch!*
*C1: Yeah! And any more mention of code status or anything else negative we're not interested in hearing.*
*P4: With her organs failing her and her increasing need for life support, I am concerned that she is actively dying.*
*S: No. We don't accept that. She won't accept that.*
*P5: Tell me what she was like before this current illness.*
*C3: You would have liked her. She made friends everywhere she went. Even if it was her first time meeting you, she could have you laughing in two minutes. She loved life. She scaled back from her previous job a few years ago, but then started working again in public relations at a health care facility. She loved what she did, and everyone she worked with loved her.*
*P6: She sounds like an amazing person. I can tell you loved her very much.*
*S: No kidding.*
*P7: If she could see herself now, in her current situation, what do you think she would say?*
*C1: She would fight. She would pray.*
*S: She never gave up. Not one day in her life. She would want nothing less than to walk with me out of here and go home.*
*C2: I know what you're saying and what you're getting at. The answer is no. We're never going to let her go. We want…She wants to fight to the end. She's going to get better and go home.*
*P8: What if she knew that going home is not an option in her near future?*
*S: You better go back and make it an option!*

Disclaimer: All identifiable information about the patient and family has been removed and/or further de-identified to protect both the patient and the family.

The verbatim exercise typically has three parts: (1) background (demographic, cultural, ethnic, and religious) information of the patient and family; (2) the full written verbatim account of the dialogue between the trainee and the patient/family; and (3) a comprehensive analysis of the verbatim following a specific set of prescribed questions for reflection. See Appendix

The verbatim patient encounter example is chosen only by the trainee (in our case the HPM fellow). We suggest the fellow choose a recent patient and/or family member encounter that was challenging or difficult. A recent patient encounter is usually fresher on the mind of the fellow and can be recreated with greater accuracy. This is often very helpful in the analysis and discussion of the verbatim with the trainers (fellowship faculty and verbatim facilitators).

The trainee (fellow) recollects and completely writes the patient and family conversation encounter word for word and then completes the entire write-up by answering a set of specifically prescribed questions.

The development of a survey was done retrospectively as we noticed the benefits of this training for our fellows, including their improved capacity for navigating difficult conversations as well as having more empathy for and connection with their patients and families. The survey (displayed as follows) asks about improvement in the areas of mindfulness related to emotions and thoughts, and the capacity for empathy during difficult patient and family encounters. The survey also asks about recognition of psychospiritual distress and about establishing effective therapeutic relationships with patients and families. The fellows were also asked for written feedback on the survey. The survey questionnaire was then implemented as an assessment tool.

## Results

### Survey responses of our last five fellows for their verbatim participation

After fellowship completion, each of our last five fellows were sent a simple six-question survey regarding the verbatim exercise. Results reflect fellowship years 2017 to 2022. Aggregate data are presented to protect the identity of our individual fellows.

The first five questions of the survey use a 5-point Likert scale fellows as follows: 1 = Strongly Disagree, 2 = Disagree, 3 = Neutral, 4 = Agree, and 5 = Strongly Agree.

Question 6 (hereunder) requested further handwritten feedback.

(The questionnaire) **Participation in the Verbatim Exercise**

1. Helped me to be more mindful of my thoughts and emotions during patient encounters.
2. Increased my feelings of empathy with patients and/or their families.
3. Made me better able to recognize psychosocial and spiritual distress.
4. Improved my ability to establish an effective therapeutic relationship with patients.
5. Was a valuable addition to my training in HPM.

Eighty percent of our surveyed fellows for the past five years agreed that the verbatim exercise was helpful in their growth as HPM practitioners. Eighty percent of our surveyed fellows felt that the verbatim helped them be more mindful of thoughts and emotions during patient encounters. Sixty percent of our fellows felt that the verbatim increased their feelings of empathy with patients and/or families. Sixty percent of our fellows felt that the verbatim better enabled them to recognize psychosocial or spiritual distress in patient encounters. Sixty percent of our fellows felt that the verbatim improved their ability to establish an effective therapeutic relationship with patients. Eighty percent of our fellows felt the verbatim was a valuable addition to the fellowship training.

Question 6 asked for written feedback responses—both positive and negative, summarized as follows.

Question 6. **Positive comment**s included: “a critical review from the outside helped me to see my own perceptions and biases from a different lens, allowing for reflection and improvement”; “this (exercise) placed me in a position to recognize my own faults and biases that I may bring into patient encounters”; and “this helped me uncover a lot of unconscious biases that may affect my decision making.” “This is a challenging exercise. “Great avenue for self-reflection and served as a vital part of my personal and professional growth this year.”

Question 6. **Constructive/Negative feedback comments** (with some paraphrasing) included: “Some fellows may not be as receptive to this exercise.” “The context of my involvement was that of remediation (author: this was this fellow's perception), therefore I had a defensive posture.” “The exercise was emotionally straining”;“it was sometime difficult to generate verbatims from memory”; and “would be good to have supportive follow-up discussion (***author's insert***—from our fellowship psychologist) for further self-discovery and support.”

## Discussion

Through our three verbatim exercises per year, our fellows learn to improve their navigation of uncomfortable feelings amidst challenging clinical patient encounters. With thorough group reflection and discussion, the fellow grows in learning to better support and empower her-/himself as well as the patient and family. Fellows are given support in recognizing their own biases and attitudes toward certain patients and/or caregivers. In this protected discussion the fellow has repeated opportunities to reflect more deeply upon the verbatim encounter, their own emotions, their spirituality, and potentially grow in self-awareness too.

Understanding our personal biases helps relieve some of the emotional pain associated with these biases and enhances one's perspective when working with “difficult” patients.^5^ Physician self-awareness is a critical skill not often taught in medical education and particularly important for resiliency in palliative medicine.^5,10^ The verbatim exercise can help promote both self-awareness and support professional growth toward a more mindful medical practice.^5–7,10^ This too supports achievement in the professionalism milestone of the HPM ACGME fellowship milestones.

Fellows are invited to see that their professional value and effectiveness in a conversation is not based on an agenda or getting a patient or family somewhere more realistic. We have seen our fellows become less emotionally burdened in allowing the process of dying to uniquely unfold. Our fellows learn that this process is never easy with complex cases or maladaptive coping strategies. We remind our fellows that clinical encounters where a family is holding on to an unrealistic outcome may be their only viable option at that time. In supporting the patient and family to navigate their own way, fellows may help them arrive in a new place of understanding of the patient's dying process. This growth in interpersonal communication supports the achievement of that ACGME HPM milestone. A third ACGME HPM milestone addressed by verbatim participation is that of practice-based learning. Fellows grow with practice, support, and feedback, and the verbatim can facilitate this growth throughout and beyond the fellowship year.

Regarding the negative feedback comments from the fellows, we observed that it was difficult for our fellows to look closely at their own personal or professional biases. And this of course tends to be true for most humankind.

Regarding safety, our residency-trained chaplains serve as our verbatim facilitators and have extensive knowledge and experience in both participation and facilitation of the exercise. Participation in the verbatim may bring emotional discomfort, which is often difficult for the fellow to navigate. That said, our fellowship has followed the chaplain residency training processes for safe and supportive performance of the verbatim.^8,17^ We have both emotional and psychological support for our fellows throughout their training year as they meet with a counseling psychologist who serves in a nonevaluative role of support as per HPM fellowship guidelines. More specifically, for any fellow in whom difficult emotional content emerges during or after a verbatim the support and safety mechanisms are already in place.

The verbatim invites the fellow to look deeply at the how and the why and the way they respond in difficult patient/family encounters. In doing so the facilitators offer guidance and support for the fellow to also consider other communication practices that can foster compassion and two-way personal and professional growth. The more the fellow knows of him-/herself, the more of that self can be accessed and available to share in patient care encounters. The fellow's growth will demonstrate the competent use of communication skills, including emotional availability, cultural humility, appropriate self-disclosure, positive use of power and authority, a nonanxious and nonjudgmental presence, and clear and responsible boundaries.^8^ These outcomes are well aligned with the goals of the three cited ACGME milestones earlier.

The verbatim also supports the development of entrustable professional activities for fellowship training.^12,13^ Specifically, EPA #'s (5) establish goals of care based on patient/family values and specific medical circumstances; (7) prevent and mediate conflict and distress over complex medical decisions; and (13) promote self-care and resilience. These three EPAs are also specifically addressed by participation in the verbatim exercise.

Limitations of our study include the fact that we only train one fellow per year, and so our sample size is small. Additional limitations included the fact that the survey data were retrospective. But we did not want the results to be influenced before the fellows had completed their fellowship year and completed the three planned verbatim exercises.

## Conclusion

In summary, the verbatim is a unique formative educational tool that promotes both personal and professional growth and supports achievement in three of the six ACGME HPM fellowship milestones: interpersonal communication, professionalism, and practice-based learning. The benefits of the verbatim exercise support a more humane and effective HPM medical practice, build resiliency, and support growth in mindfulness.^5,6,9,10,16^ These qualities protect the HPM practitioner against burnout, which is essential in the field of palliative medicine.^9–11^

More research is needed to determine the impact of the verbatim on empathic responses from trainees as well as the specific achievement of the ACGME HPM milestones.

## Abbreviation Used

HPM: Hospice and Palliative Medicine

## Appendix. Anatomy of Verbatim Exercise

For each verbatim written, our fellow identified an encounter with a recent patient and/or family member(s) that would serve for review and discussion. Each fellow is encouraged to share a challenging case. Verbatims typically involve three main sections:

1. Demographic, cultural, ethnic, and/or religious background of the patient, as well as the full context of the encounter.
2. The “verbatim” account of the dialogue between the fellow and the patient and/or family member(s), representing the trainee's best memory account of what was said and observed during the encounter.
3. A comprehensive analysis of the verbatim writer's self-reflection, including strengths, weaknesses, and overall connection between patient and/or family member(s). A set of standard questions guides the verbatim writer.

As the fellow and the teachers/supervisors come together, the verbatim is reviewed thoroughly in the following manner:

1. Fellow reads initial information and demographics of the patient.
2. The verbatim is read by the other participants (teaching faculty/supervisors) taking various roles in the encounter (such as patient, doctor, and family member). The fellow listens whereas others role-play the full encounter, including the patient and/or family.
3. Fellow then reads their written reflective evaluation sections.
4. Facilitators and the fellow address key messages for each person in the verbatim.
5. Facilitators and the fellow address key dynamics happening between each person.
6. During feedback discussion, facilitators and the fellow identify strengths and growing edges of the encounter, as well as provide further insight and encouragement for future encounters.
7. Each participant identifies at least one thing learned during the session.

Common questions from chaplain and physician participants to the fellow include some of the following:

A. With whom do you most closely identify?
B. How are you like this person with whom you had difficulty?
C. Are you able to acknowledge [identified emotion] in this encounter?
D. What did you imagine the patient/family were thinking and feeling?
E. What does this encounter say about you?
F. How has this verbatim affected your practice?
G. If you could do it all over again, what would you do or say differently if the same scenario presented itself?
